# Are Australian clinicians monitoring medication adherence in hematological cancer survivors? Two cross-sectional studies

**DOI:** 10.1186/s40164-015-0011-4

**Published:** 2015-06-11

**Authors:** Marita C. Lynagh, Tara Clinton-McHarg, Alix Hall, Rob Sanson-Fisher, William Stevenson, Campbell Tiley, Alessandra Bisquera

**Affiliations:** School of Medicine and Public Health, Faculty of Health and Medicine, University of Newcastle, Level 4, West, HMRI Building, Callaghan, NSW 2308 Australia; School of Medicine and Public Health, Faculty of Health and Medicine, The University of Newcastle, 1127 Booth Building, Wallsend Campus, NSW 2308 Australia; Department of Haematology Royal North Shore Hospital Pathology North, The University of Sydney, Sydney, NSW 2006 Australia; The Clinical Research Design Information Technology and Statistical Support Unit (CReDITSS), The University of Newcastle, HMRI Building, Callaghan, Sydney, NSW 2308 Australia

**Keywords:** Medication adherence, Hematological cancer, Oral therapy

## Abstract

**Background:**

Hematological cancer survivors are growing in number and increasingly rely on oral therapy. Given known poor outcomes associated with non-adherence and previous evidence that many patients do not fully adhere to their treatment regimen, this study aimed to determine the degree to which clinicians monitor adherence to oral medication in hematological cancer survivors.

**Methods:**

Data was combined from two cross-sectional surveys of a heterogeneous sample of 431 hematological cancer survivors recruited from three outpatient hematology clinics in three different states (*n* = 215) and one state cancer registry (*n* = 216) in Australia. Participants completed a self-administered survey that included demographic characteristics and a 7-item measure of medication adherence developed by the researchers specifically for the purpose of the studies.

**Results:**

Of the 431 participants, 37 % (*n* = 160) reported currently taking daily cancer-related medication. Of these, 14 % (*n* = 23) were found to be non-adherent with ‘missing a dose’ being the most commonly reported non-adherent behaviour. Only 41 % of survivors indicated that their hematologist or cancer clinician had ‘always’ asked about their cancer-related medication during their last six visits.

**Conclusions:**

Non-adherence to oral therapy remains a problem in hematological cancer survivors, yet clinicians in Australia do not appear to be regularly monitoring adherence in their patients. Given an increasing dependence on oral therapy in clinical hematology and medical oncology and the importance of medication adherence to optimising health outcomes, greater effort should be invested in developing effective interventions to improve support and adherence monitoring by cancer clinicians and GPs.

## Background

Patient non-adherence to medication is a well-recognised problem in all cancer populations [1], including among hematological cancer patients [2]. Recent findings from three different studies on adherence to imatinib oral therapy in Chronic Myeloid Leukaemia (CML) patients confirm this phenomenon [3–5]. The proportion of patients not adhering to medication is estimated to be 25 % using pharmacy and medical claims data [3], 36 % using self-report and pill counts [4] and 2 % using MEMS (microelectronic monitoring devices) [5]. Despite median non-adherence rates being low, Marin and colleagues found that 26 % of patients were less than 90 % adherent and 14 % of patients were less than 80 % adherent [5].

Poor medication adherence among hematological cancer patients plays a significant role in treatment failure [6] and survival [7]. For example, patients with lower imatinib adherence rates have poorer responses to therapy (such as loss of cytogenetic responses) and higher medical costs [3–5]. Given that: 1) the number of hematological cancer survivors is growing [8]; 2) complex and lengthy treatment regimens [9] are becoming increasingly reliant on oral therapy; and 3) serious adverse outcomes have been associated with poor drug adherence, regular assessment of medication adherence by health care providers in this population is critical.

To date, a range of factors involving the patient, their disease characteristics, clinicians and the health care system [10, 11] have been found to impact on medication adherence. These factors include: treatment side effects [10, 12]; poor patient-doctor relationship [13]; and medication costs [13]. Patients who lack knowledge about the side-effects and benefits of medication [14]: feel less control over their health care [15]; dislike aspects of their medication [15]; have a poorer disease prognosis [16]; and perceive inadequacies in their health care delivery [16], are also less likely to adhere. Having insight into these factors may assist clinicians in recognising patients who may be at greater risk of not adhering to oral therapy.

While disease progression, complications or lack of response to therapy may all be signs of poor adherence, these may nevertheless be absent in a patient who is not taking oral medication as prescribed. A key component to improving medication adherence is follow-up and support [17]. Patients should be regularly asked, in a non-judgmental way, about their use of medications by their clinicians [10, 17]. However, a review of the field has found no studies to date which have investigated the degree to which clinicians ask cancer survivors about their adherence to oral therapy.

Further, although hematological cancer survivors are a diverse group, the few studies investigating medication adherence published to date have focussed predominantly on adherence in CML patients only and report wide variations in adherence rates [3–5]. There remains a vital need to investigate the problem of medication non-adherence in more heterogeneous hematological cancer populations to identify the most common patterns of non-adherence behaviours and better understand clinician practice so that effective strategies can be employed to improve adherence among survivors.

The purpose of this study was to assess self-reported medication adherence by hematological cancer survivors and perceived clinician behaviour in relation to monitoring of their oral therapy adherence. Specifically, it aimed to:

1. Identify the proportion of hematological cancer survivors in Australia who report non-adherence to medication;
2. Describe the frequency with which Australian cancer survivors report being asked about their medication adherence by their hematologists or cancer doctors, and their general practitioners (GPs) or family doctors;
3. Identify demographic and disease characteristics of survivors associated with infrequent medication adherence monitoring by clinicians.

## Method

### Study design & setting

The study combined data from two independent cross-sectional surveys of adult hematological cancer survivors. In one study, survivors were recruited from three outpatient hematology treatment clinics in three different Australia states, while the second study recruited survivors from one state cancer registry in a fourth Australian state. For both studies, the data presented here on medication adherence was one component of a much larger national study on the unmet needs of haematological cancer survivors.

### Sample and procedure

Eligible participants were aged 18 years or older at time of the studies, could read and understand English, and had a confirmed diagnosis of hematological cancer. For registry-recruited participants, diagnosis was within the last 3 years.

At treatment clinics, a hematologist or nurse first identified potentially eligible patients who were then approached by a research assistant and invited to take part in the study from October 2012 to March 2013. Consenting patients were asked to complete two pen and paper questionnaires, one in the clinic while waiting for their appointment (which included demographic and disease characteristics) and a second questionnaire one month later that was posted to their home address (containing the medication adherence questions). One written reminder was sent to patients who had not returned their completed survey within two weeks of consenting to participate. Three hundred and ninety-five (395) patients were identified as eligible and approached to participate in the study, and 353 (89 %) consented to participate.

A rolling recruitment method was utilised at the cancer registry. Eligible survivors were identified and approached on an on-going basis between September 2012 and September 2013. Active clinician consent was employed as the standard patient recruitment procedure by this registry. This meant that consent was first required from the treating clinician before the registry contacted their patients. Contacted patients were asked if they agreed to have their contact details released by the registry to the researchers. Participants who consented to having their contact details released were sent a self-administered pen-and-paper questionnaire. Non-responders were mailed a second copy of the survey approximately four weeks later. There were 1480 hematological cancer survivors identified as eligible for the study. Clinicians consented to the registry contacting 616 of these, with missing registry data, clinician non-response and deceased participants being the main reasons for clinician non-consent. Of the 616 survivors approached by the registry, 316 (51 %) consented to having their contact details forwarded to the researchers.

Ethics approval was obtained from the University of Newcastle Human Research Ethics Committee and the relevant ethics committees associated with each of the treatment clinics and the cancer registry.

### Measures

Participants completed a self-administered questionnaire that included questions about demographic characteristics (eg. marital status, education level, employment status) and cancer history and treatment. For participants recruited via the cancer registry, some demographic and disease characteristics (eg. sex, postcode, country of birth, cancer type) were obtained directly from the cancer registry’s records.

Participants also completed seven items assessing medication adherence and perceived clinician behaviour that were developed by the research team in close consultation with hematologists and oncologists. Relevant medication adherence behaviours were first identified from a review of the literature, checked against behaviours reported as important by clinicians, and then arranged into a draft questionnaire. The draft questionnaire was sent to three hematologists in Australia and three international experts in the field of medication adherence for review. Any possible missing medication adherence behaviours identified by the experts were then added to the questionnaire. Item wording and response options were also refined. A copy of the final seven items used can be seen in Table 1.

**Table 1:** Seven items assessing Medication Adherence & Clinician Behaviour

Thinking about the prescribed medications you are taking related to your cancer or treatment side-effects:			
1. How many different prescribed medications related to your cancer or treatment side-effects are you currently taking EACH DAY?	Nil / 1–2 / 3–4 / 5–6 / 7–8 / 9 or more		
2. In the last 7 days, have you missed a dose of one or more of your medications?	No	Yes	
3. In the last 7 days, have you taken a medication at the wrong time? (eg. taken the medication at lunchtime instead of with breakfast)	No	Yes	
4. In the last 7 days, have you taken a higher dose of a medication than as prescribed?	No	Yes	
5. In the last 6 months, have you stopped taking any prescribed cancer-related medications without first getting your doctor’s approval to do so?	No	Not sure	Yes
6. At your last 6 visits to your hematologist or cancer doctor, on how many of these occasions did they ask you whether you have been taking your cancer-related medications as prescribed?	Always, at every appointment		
	Sometimes, but not at every appointment		
	Never		
	Not sure / can’t remember		
7. At your last 6 visits to your GP or regular doctor, on how many of these occasions did they ask you whether you have been taking your cancer-related medications as prescribed?	Always, at every appointment		
	Sometimes, but not at every appointment		
	Never		
	Not sure / can’t remember		

### Statistical analysis

For the purpose of this study, ‘non-adherence’ was defined as reporting at least one of the four behaviour patterns associated with non-adherent behaviour (items 2 to 5 in Table 1). Statistical analysis was undertaken using SAS version 9.4 (SAS Institute Inc, Cary, NC). For each of the seven items assessing adherence and clinician behaviours, the frequency and percentage of responses were calculated to identify the proportion of hematological cancer survivors who reported non-adherence to medication, and the frequency with which cancer survivors report being asked about their medication adherence by their hematologists or their general practitioners (GP)/family doctors. Fisher’s exact analyses were used to compare medication adherence behaviour by sex, age, rural versus urban geographical location and cancer type. Rural versus urban location was defined using postcode at diagnosis, based on the Australian Bureau of Statistics (ABS) five categories of the Accessibility and Remoteness Index of Australia (ARIA+) classification [18]. Participants whose postcodes fell within the ABS categories of major cities of Australia and inner regional were classified as ‘urban’. Those with postcodes falling within the categories of outer regional, remote and very remote Australia were defined as ‘rural’. Fisher’s exact test was also used to identify demographic and disease characteristics associated with infrequent medication adherence monitoring by clinicians. While sample size estimates were not calculated for the sub-studies presented, both of the larger studies from which they derived were estimated to permit statistical analyses with a power of 80 % and a significance level of 5 % in relation to the main outcome measures.

## Results

### Participants

Two hundred and fifteen patients attending outpatient hematological treatment centres and 216 hematological cancer survivors recruited through the cancer registry completed survey items pertaining to medication adherence (total *n* = 431), representing participation rates of 54 % and 42 % respectively. Of these, 160 participants (37 %) reported taking at least one medication each day related to their cancer diagnosis or treatment side-effects. The majority of participants were males (55 %) and only 3 % of participants were aged less than 40 years (see Table 2). The most common cancer type was myeloma (38 %). Participants recruited through treatment centres differed from those recruited via the cancer registry in regard to usual residence (*p* < 0.0001) and time since diagnosis (*p* < 0.0001) with treatment centre participants more likely to live in urban areas and be less than 2 years post-diagnosis compared with registry-recruited cancer survivors. Participants differed from non-participants in regard to age (*p* = 0.0007) with fewer participants aged below 50 years and cancer type (*p* = 0.029) with higher proportions of participants with NHL and myeloma and a lower proportion of Hodgkin lymphoma cancer patients. The study samples did not differ from non-participants by sex or usual residence. The 160 participants who reported taking daily medication differed from non-medication participants in time since diagnosis (*p* < 0.0001) with medicated participants more likely to be diagnosed less than 2 years prior, and by cancer type (*p* < 0.0001) with higher proportions of participants with acute leukaemia and myeloma reporting taking cancer-related medications every day.

**Table 2:** Demographic and disease characteristics of participants^a^

Characteristic		Cancer Registry participants (*N* = 62)	Treatment Centre participants (*N* = 98)	Total (*N* = 160)	Exact *p*-value
		*n* (%)	*n* (%)	*n* (%)	
Sex^b^	Male	26 (49 %)	57 (58 %)	83 (55 %)	0.307
	Female	27 (51 %)	41 (42 %)	68 (45 %)	
Age^b^	18–39 years	1 (2 %)	4 (4 %)	5 (3 %)	0.658
	40–49 years	5 (9.4 %)	7 (7 %)	12 (8 %)	
	50–59 years	18 (34 %)	25 (26 %)	43 (28 %)	
	60–69 years	12 (23 %)	31 (32 %)	43 (28 %)	
	70 years and over	17 (32 %)	31 (32 %)	48 (32 %)	
Cancer type^b^	Non-Hodgkin Lymphoma (NHL)	20 (38 %)	18 (18 %)	38 (25 %)	0.051
	Acute Leukaemia (AL)	8 (15 %)	11 (11 %)	19 (13 %)	
	Chronic Lymph Leukaemia (CLL)	3 (6 %)	11 (11 %)	14 (9 %)	
	Myeloma	19 (36 %)	39 (40 %)	58 (38 %)	
	Hodgkin Lymphoma (HL)	1 (2 %)	6 (6 %)	7 (5 %)	
	Other	2 (4 %)	13 (13 %)	15 (10 %)	
Usual residence^b^	Urban	30 (57 %)	95 (97 %)	125 (83 %)	<0.0001
	Rural	23 (43 %)	3 (3 %)	26 (17 %)	
Time since diagnosis^b^	Less than 2 years	-	52 (53 %)	52 (34 %)	<0.0001
	2 years and over	53 (100 %)	46 (47 %)	99 (66 %)	
Marital status^b^	Married/living with partner	51 (84 %)	71 (74 %)	122 (78 %)	0.174
	Single, never married	10 (16 %)	25 (26 %)	35 (22 %)	
Education^b^	High school or below	28 (48 %)	45 (50 %)	73 (49 %)	0.818
	Trade or TAFE	15 (26 %)	26 (29 %)	41 (28 %)	
	University degree	15 (26 %)	19 (21 %)	34 (23 %)	
Employment^b^	Currently employed	23 (38 %)	32 (33 %)	55 (35 %)	0.607
	Not employed	38 (62 %)	66 (67 %)	104 (65 %)	

^a^Participants are those who reported currently taking daily cancer-related medications

^b^Totals may not equal sample size due to missing data values

### Medication non-adherence behaviour of hematological cancer survivors

**Fig. 1:**
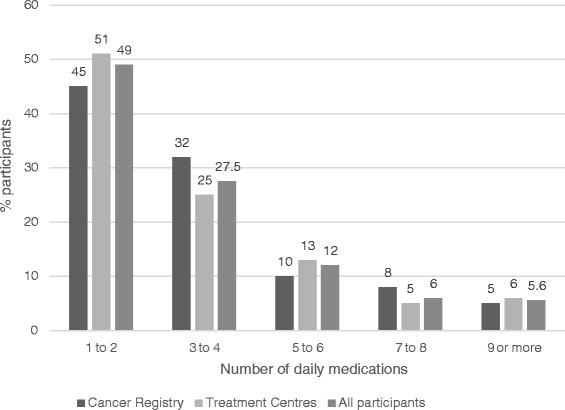
Number of daily prescribed cancer-related medications reported by hematological cancer survivors recruited for each study

Of the 160 participants taking daily medications, almost a quarter (24 %, *n* = 38) were taking five or more medications each day (see Fig. 1). Eighty-six percent of participants prescribed oral therapy were found to be adherent, with 14 % (*n* = 23) defined as ‘non-adherent’ according to the study criteria. The most commonly reported non-adherent behaviour was missing a dose (9 %, *n* = 15) with very few participants failing to take their medication at the correct time (3 %, *n* = 4) or ceasing a medication without their doctor’s approval (3 %, *n* = 4) (see Table 3). Usual residence was significantly associated with non-adherence behaviour with urban participants more likely to be non-adherent compared with rural (*p* < 0.007). The only other factor significantly associated with non-adherence was age with those aged less than 40 years more likely to report non-adherence behaviours (*p* < 0.039).

**Table 3:** Medication non-adherence behaviour in hematological cancer survivors

	Participants (*N* = 160)	
Medication non-adherence behaviours	N	%
Missed a dose of 1 or more medications in last 7 days	15	9
Did not take a medication at the correct time prescribed in last 7 days	4	3
Took a higher dose of medication than prescribed in last 7 days	3	2
Stopped taking a prescribed medication without first getting doctor’s approval in last 6 months	4	3
Reported at least 1 or more medication non-adherence behaviour	23^a^	14

^a^ Some participants reported more than one behaviour

### Monitoring of medication adherence by clinicians

Among hematological cancer survivors on daily oral therapy, only 41 % indicated that their hematologist or cancer doctor ‘always’ asked whether they had been taking their prescribed cancer-related medications during their last six visits. Almost a quarter (23 %, *n* = 37) of participants reported that they were ‘never’ asked about their medications. A high proportion of participants (40 %) also indicated that they were never asked whether they had been taking their prescribed cancer-related medications by their GP or regular doctor during the last six visits, with only 29 % reporting being ‘always’ questioned about cancer-related medications by their GP (see Table 4). There was no significant difference in adherence behavior between participants who reported “always” being asked by their hematologist or their GP about medications compared to those who reported that they were asked only “sometimes” or “never” (*p* = 0.072).

**Table 4:** Clinician assessment of medication adherence in hematological cancer survivors (N = 160)

Clinician behaviour	Always *N* (%)	Sometimes *N* (%)	Never *N* (%)	Not sure/can’t remember *N* (%)
Hematologist or cancer doctor asked whether you have been taking cancer-related medications as prescribed during last 6 visits^a^	64 (41 %)	48 (30 %)	37 (23 %)	9 (6 %)
GP or regular doctor asked whether you have been taking cancer-related medications as prescribed during last 6 visits^b^	45 (29 %)	42 (27 %)	63 (40 %)	7 (4 %)

^a^Missing data *n* = 2

^b^Missing data *n* = 3

### Factors associated with clinician monitoring of adherence

For both hematologists and regular GPs, no significant differences were found between cancer survivors who were ‘always’ asked about taking their cancer-related medication and those who were not, in relation to the survivors’ gender, age, cancer type, time since diagnosis, marital status, education level and number of medications taken per day (see Table 5).

**Table 5:** Associations between patient characteristics and clinician monitoring of medication adherence

		Monitoring by Hematologist			Monitoring by GP or regular doctor		
Characteristic		Always (*n* = 64)	Sometimes or never (*n* = 94)	Exact *p* value	Always (*n* = 45)	Sometimes or never (*n* = 112)	Exact *p*-value
		*n* (%)	*n* (%)		*n* (%)	*n* (%)	
Sex^a^	Male	30 (48 %)	52 (60 %)	0.185	21 (48 %)	61 (59 %)	0.278
	Female	32 (52 %)	35 (40 %)		23 (52 %)	43 (41 %)	
Age^a^	18–39 years	2 (3.2 %)	3 (3.4 %)	0.655	2 (4.5 %)	3 (2.9 %)	0.878
	40–49 years	7 (11 %)	5 (5.7 %)		4 (9 %)	8 (7.7 %)	
	50–59 years	19 (31 %)	24 (28 %)		14 (32 %)	28 (27 %)	
	60–69 years	18 (29 %)	25 (29 %)		12 (27 %)	30 (29 %)	
	70 years and over	16 (26 %)	30 (34 %)		12 (27 %)	35 (34 %)	
Cancer type^a^	Non-Hodgkin Lymphoma	16 (26 %)	21 (24 %)	0.599	14 (32 %)	22 (21 %)	0.253
	Acute Leukaemia	7 (11 %)	12 (14 %)		3 (6.8 %)	16 (15 %)	
	Chronic Lymph Leukaemia	7 911 %)	7 (8 %)		3 (6.8 %)	11 (11 %)	
	Myeloma	22 (35 %)	35 (40 %)		17 (39 %)	40 (38 %)	
	Hodgkin Lymphoma	5 (8 %)	2 (2.3 %)		4 (9 %)	3 (2.9 %)	
	Other	5 (8 %)	10 (11 %)		3 (6.8 %)	12 (12 %)	
Usual residence^a^	Urban	52 (84 %)	72 (83 %)	1.000	37 (84 %)	86 (83 %)	1.000
	Rural	10 (16 %)	15 (17 %)		7 (16 %)	18 (17 %)	
Time since diagnosis^a^	Less than 2 years	24 (39 %)	28 (32 %)	0.486	20 (45 %)	31 (30 %)	0.088
	2 years and over	38 (61 %)	59 (68 %)		24 (55 %)	73 % (70 %)	
Marital status^a^	Married/living with partner	17 (27 %)	18 (20 %)	0.336	13 (29 %)	22 (20 %)	0.291
	Single, never married	47 (73 %)	73 (80 %)		32 (71 %)	87 (80 %)	
Education^a^	High school or below	27 (45 %)	44 (51 %)	0.309	19 (46 %)	53 (51 %)	0.786
	Trade or TAFE	21 (35 %)	20 (23 %)		13 (32 %)	27 (26 %)	
	University degree	12 (20 %)	22 (26 %)		9 (22 %)	24 (23 %)	
Employment^a^	Currently employed	23 (36 %)	32 (34 %)	0.866	16 (36 %)	38 (34 %)	1.000
	Not employed	41 (64 %)	61 (66 %)		29 (64 %)	73 (66 %)	
No. of daily medications	2 or less	31 (48 %)	46 (49 %)	1.000	21 (47 %)	56 (50 %)	0.727
	3 or more	33 (52 %)	48 (51 %)		24 (53 %)	56 (50 %)	

^a^Totals may not equal sample size due to missing data values

## Discussion

Although many hematological cancers require long-term management [9], this study found that only 37 % of participants were taking daily medication related to their cancer or side-effects of treatment. As a significant proportion of participants had been diagnosed more than two years prior to the survey, it is likely that some may have completed treatment and/or were in the ‘watchful waiting’ stage of survivorship [19].

Of the 160 survivors who were currently prescribed oral therapy, the majority (86 %) were found to be adherent. While this provides evidence that medication non-adherence among hematological cancer survivors occurs, it suggests that the problem may not be as extensive as previous research has indicated [11]. Our self-reported non-adherence rate of 14 % is less than the 36 % found by Noens et al. [4] but greater than the 9 % reported in the findings of the IRIS trial (International Randomized Study of Interferon versus ST1571) [20]. The difference in reported non-adherence rates could be due to the fact that previous studies had investigated only CML patients in contrast to the present study which included all major hematological cancers. Further, it is likely that the 9 % non-adherence rate reported in the IRIS clinical trial [2] was due to the close surveillance and greater assessment frequency of patients involved in a clinical trial compared to “real world” patient populations.

The most commonly reported non-adherent behaviour in the current study was missing a dose, consistent with broader findings relating to medication adherence for general health care [21]. However, we were not able to determine whether this behaviour was unintentional (ie. simply forgetting) or a deliberate choice to not take a medication. Previous research has suggested that few cancer patients intentionally choose to miss a dose [15]. Participants were further not asked to distinguish between oral cancer medications and medications taken to prevent or manage treatment side effects, hence it is possible that when a dose is missed, this could relate to medications for side-effects.

To our knowledge, this study was the first to examine clinician behaviour in relation to monitoring medication adherence in hematological cancer survivors. Only 41 % of survivors (currently taking oral therapy) reported being always asked about their medications by their cancer doctor, with most participants indicating that they were never or only ‘sometimes’ prompted by either their hematologist or regular clinician to discuss their cancer-related medications. Even though survivor self-report was used as a proxy measure for actual clinician behaviour, the perceived low frequency of clinician-initiated questions regarding medications may be interpreted by cancer survivors to mean that medications and adherence to a prescribed schedule are not important to their prognosis. Given evidence that patients’ beliefs about the costs and benefits of oral therapy is a strong predictor of adherence [22, 23], further research is needed to investigate the actions of clinicians and their role in guiding survivors’ beliefs about the risk-benefit ratio. Clinicians themselves acknowledge the critical role they play in patient education [4] and endorse the routine assessment of adherence behaviour in hematological cancer survivors [4, 10]. The perceived poor assessment of medication adherence by clinicians may reflect an assumption on the clinician’s behalf that patients are fully adherent or a belief that survivors with a longer time since diagnosis may have already developed a good understanding of the importance of taking their medication.

### Strengths and limitations

This study investigated a diverse sample of hematological cancer survivors recruited from two different sources as opposed to focussing on only a single hematological cancer type or recruiting patients via treatment centres only. Many participants had already gone through intensive treatment and were currently focussed on the long-term management of their cancer, providing a unique and previously unexplored hematological cancer population. Despite these strengths, the findings of this study should be considered within the limitations of the data and study design. First, adherence was measured using participant self-report which may over or under-estimate medication adherence compared to other direct and/or indirect measurement methods [10]. However, in the absence of a gold standard, patient self-report is still considered to be the most simple and cost-effective measurement method [10]. Second, as previously noted, clinician monitoring behaviour was also measured via patient self-report rather than direct observation or clinician self-report and as such may be subject to recall bias. Third, the combined study samples recruited via two different methods may limit the generalizability of findings. Given that most participants were more than two years post-diagnosis and currently taking few medications, the results obtained may not be applicable to all hematological cancer patients, particularly those who are more recently diagnosed. Further, the low consent rate of participants recruited from the registry (36 %) may also affect generalisability of results, however the response rate in the present study is comparable with other studies utilising cancer registries for recruitment [24, 25]. Finally, the present study only investigated monitoring of adherence by hematologists and regular doctors. Pharmacists are increasingly playing a critical role in managing medication adherence in patients but were not included in the present study [17]. While we attempted to identify whether there were any specific patient characteristics associated with clinician monitoring of adherence, other potential characteristics of clinician behaviour (such as clinician demographics and health care system factors) were not measured. Further research should explore these and other variables to provide a greater understanding of barriers to medication adherence monitoring by clinicians.

## Conclusion

This study has shown that a small but significant proportion of hematological cancer survivors do not adhere to their oral therapy regimen. Missing a dose of one or more medications in the last seven days was the most commonly reported non-adherent behaviour. Despite the serious consequences associated with non-adherence to oral therapy and acknowledgement of the need to provide follow-up support to survivors, the findings presented here indicate that clinicians are not routinely monitoring adherence, or at least survivors are not recalling the occurrence of such monitoring. Future research should focus on the development of interventions to support and improve adherence monitoring by cancer clinicians and GPs.
